# T2-mapping- influence of arrhythmia and heart rate: A phantom experiment

**DOI:** 10.1186/1532-429X-18-S1-W36

**Published:** 2016-01-27

**Authors:** Marcel Prothmann, Matthias A Dieringer, Jeanette Schulz-Menger

**Affiliations:** Cardiology, Charité. Medical Faculty of Humboldt-University Berlin ECRC and HELIOS Clinics, Berlin, Germany

## Background

Myocardial tissue differentiation applying Cardiovascular magnetic resonance (CMR) offers the capability to detect myocardial injury already in preserved ejection fraction. Detection of edema is a unique capability of CMR. Conventional T2 imaging suffers from technical challenges related to diseases specific issues like tachycardia, atrial fibrillation and pericardial effusion. T2-mapping seems to be more stable based on visual assessment. Little is known about its dependency of disease relate abnormalities. We aimed to investigate on the influence of heart rhythm abnormalities on T2 mapping.

## Methods

**Figure 1:**
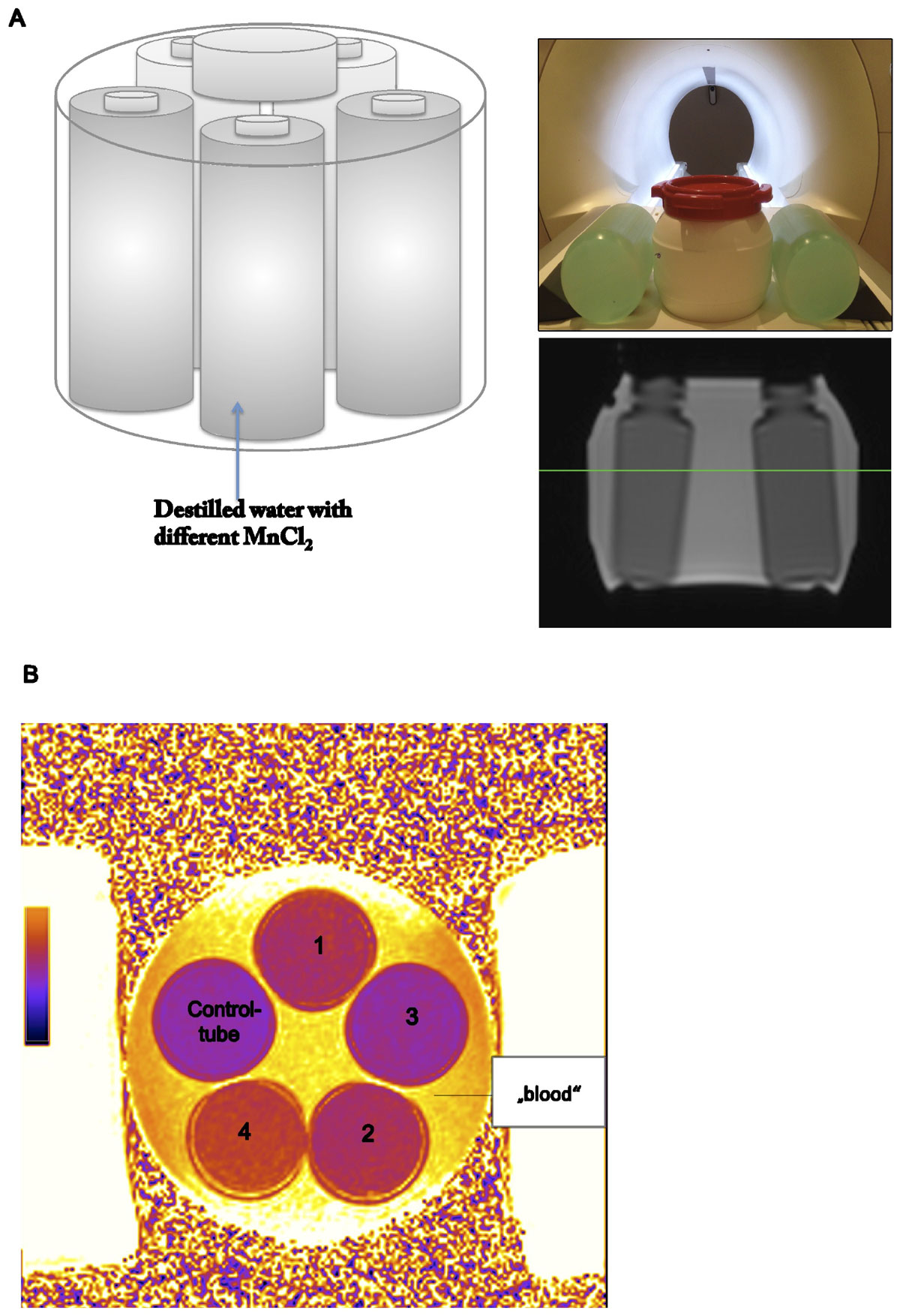
**A - shows the construction of the T2 phantom B - T2map Phantom**. Each tube provides different T2-time.

We performed phantom experiments using a 1.5 Tesla Siemen Avanto Fit (Siemens Healthcare, Erlangen, Germany) equipped with a 32-channel cardiac coil. The T2 phantom consisted of five tubes (figure 1A). They were filled with different concentrations of manganese(II) chloride, to achieve various T2-times (60-80 ms). Tube 1 was set to 65 ms, tube 2 (70 ms), tube3 (60 ms), tube3 (80 ms) and tube 4 (control tube 65 ms) and the solution around the tubes ("blood" =150 ms).The phantom was surrounded by two NISO_4_ + NaCL doped water bottles to achieve enough load for MRI. We applied a T2 prepared SSFP- based T2 mapping technique with the following parameters: voxel size: 1.6 × 1.6 × 6 mm³, TR 2.7 ms, flip angle 70°, TE 1,15 ms; GRAPPA reduction factor 2. Sinus rhythm heart rates of 60, 80,100 and 120 bpm were simulated using an ECG simulator (Siemens Healthcare). Arrhythmia was simulated using a commercial ECG simulation device (ES 300, SPI electronics). To assess repeatability of the T2 times, the T2 maps were acquired 6 times for each heart rate and rhythm. T2 times were quantified based on the inline T2 maps by drawing a ROI covering the entire phantom intersection using cvi42 5.0 (Circle Cardiovascular Imaging Calgary,Canada).

## Results

T2 times were evaluable in all settings. T2 times did not show significant differences depending on heart rate variability within each tube (tube1 p = 0.541; tube2 p = 0.661; tube3 p = 0.51; tube4 p = 0.813; tube5 p = 0.729). Details are given in table 1. Figure 1B shows the T2 phantom using a simulated sinus rhythm at 60 bpm.

**Table 1:** Mean T2-times (ms) depending on heart rate and rhythm

Heart rate bpm	SR 60	SR 80	SR 100	SR 120	Afib 120
Tube1	64.9 ± 0.6	63.9 ± 0.7	63.9 ± 0.9	64.5 ± 1.0	62.1 ± 2.8
Tube2	70.8 ± 0.97	70.7 ± 0.8	70.7 ± 0.8	70.9 ± 0.8	70.3 ± 0.5
Tube3	61.6 ± 0,98	61.4 ± 1.1	61.4 ± 1.1	60.8 ± 0.9	61.5 ± 1.3
Tube4	82.6 ± 1.57	81.6 ± 0.8	81.6 ± 0.8	81.5 ± 1.3	81.7 ± 3.2
„blood\"	158.5 ± 2.06	156.8 ± 3.0	156.8 ± 3.	158.1 ± 3.2	158.2 ± 2.9

## Conclusions

The influence of heart rhythm abnormalities and heart rate on T2-times seems to be not relevant. That gives the possibility to identify myocardial edema also in patients with arrhythmia. Further patient studies are needed.

